# Demographic and Clinical Characteristics of Patients Presenting With Angiotensin-converting Enzyme Inhibitors Induced Cough

**DOI:** 10.7759/cureus.5624

**Published:** 2019-09-11

**Authors:** Fatima Jamshed, Hassam Jaffry, Hamza Hanif, Vinesh Kumar, Uruja Naz, Mehak Ahmed, Sundus Fareed

**Affiliations:** 1 Pediatrics, Jinnah Sindh Medical University, Karachi, PAK; 2 Internal Medicine, Jinnah Postgraduate Medical Center, Karachi, PAK; 3 Internal Medicine, Jinnah Sindh Medical University, Karachi, PAK; 4 Internal Medicine, Ghulam Mohammad Mahar Medical College, Sukkur, PAK; 5 Pathology, Liaquat College of Medicine and Dentistry, Darul Sehat Hospital, Karachi, PAK; 6 Medicine, Liaquat College of Medicine and Dentistry, Karachi, PAK; 7 Internal Medicine, Civil Hospital Karachi, Karachi, PAK

**Keywords:** angiotensin-converting enzyme inhibitor, enalapril, ace inhibitor induced cough, cough, captopril, lisinopril, ramipril

## Abstract

Introduction

Angiotensin-converting enzyme inhibitors (ACEi) induced cough is still the greatest challenge in the continued utilization of ACEi for management of hypertension. The clinical pattern and related risk factors predisposing patients to ACEi-induced cough have not been studied in Pakistani hypertensive patients as yet. Hence, this study was conducted.

Methods

In this prospective, observational study individuals of both genders, of age 18 years or more, known cases of hypertension, taking ACEi, and having a chronic cough (>14 days) without any respiratory etiology were included. Their demographic and clinical characteristics were recorded. All data were managed using SPSS for Windows version 20.0 (IBM Corp., Armonk, NY).

Results

Enalapril was the most frequently prescribed ACEi (n=58; 47.2%) in patients with ACEi-induced cough followed by captopril (n=28; 22.7%), lisinopril (n=23; 18.7%), and ramipril (n=14; 11.4%). Higher body mass index (p=0.002), smoking (p=0.008), and longer time from the start of ACEi to the occurrence of cough (p=0.04) were the significant determinants of ACEi-induced cough. There were 33 (26.8%) participants who planned to request their physician to prescribe them an alternate medication due to their cough.

Conclusions

Enalapril was the most commonly ACEi prescribed among patients with ACEi-induced cough. Higher BMI, smoking, and longer time from the start of ACEi to the occurrence of cough related to ACEi-induced cough.

## Introduction

Angiotensin-converting enzyme inhibitors (ACEi) are indicated in the management of hypertension, heart failure, and diabetic nephropathy in both young and elderly patients [1]. Most patients tolerate ACE inhibitors well, but around 30% can develop adverse reactions like symptomatic hypotension and dry cough, however, more severe adverse reactions, including angioedema and renal failure, can also occur [2-4].

ACEi act by inhibiting the conversion of angiotensin I to angiotensin II which is a vasoconstrictive agent. ACEi also increase the levels of bradykinin which increases peripheral vascular permeability and causes vasodilatation. This has also been proposed as the mechanism behind ACEi-induced cough which is mediated by vasoactive kinins (bradykinin) and substance P [5]. However, a recent report from the Swedish population has concluded otherwise. In their genome-wide association study on ACEi-induced cough, genetic variation rather than bradykinin pathways was found to be the potential culprit [6]. ACEi-induced cough is still the greatest challenge in the continued utilization of ACEi for management of hypertension.

The incidence of ACEi-induced cough is 2.5-11% in the West, with a discontinuation rate of 4% [7, 8]. In Japanese patients with class I and II congestive heart failure taking enalapril, the incidence of a drug-induced cough has been reported to be as high as 28% [9]. In Pakistani patients, the overall incidence of dry cough was 7%. The incidence of cough was 5.5% with perindopril, 7% with ramipril, and 9% with lisinopril [10]. Older age, female gender, smoking, East Asian ethnicity, ACEi naivety, and history of cough due to another ACEi have been associated with a higher incidence of ACEi-induced cough [11, 12]. The clinical pattern and related risk factors predisposing patients to ACEi-induced cough have not been studied in Pakistani hypertensive patients as yet. Hence, this study was conducted.

## Materials and methods

A prospective, observational study was conducted with patients attending the clinic of their physicians a tertiary care hospital in Pakistan. After attaining informed consent, patients were requested to participate through a structured questionnaire from 1st June till 31st December 2017. The study was approved by the institutional review board.

The inclusion criteria comprised of individuals of both genders, of age 18 years or more, with known cases of hypertension, taking ACEi for their blood pressure (BP), and having a chronic cough (>14 days). Only patients who met all of the inclusion criteria were included. It was ensured that the cough was not related to any respiratory etiology. Patients with current respiratory tract infection (RTI), and/or pneumonia, or those who have had an episode of RTI in the last month were excluded. Known cases of asthma, chronic obstructive pulmonary disease, and/or any other chronic respiratory disease were excluded. Newly diagnosed cases of hypertension were not included.

All participants who fulfilled the inclusion criteria and also provided informed consent were requested to fill in a structured questionnaire. It comprised of their baseline information including age, gender, body weight, height, smoking status, and comorbidity status. ACEi related characteristics included type, time of the day, and duration of ACEi usage and time from the start of ACEi to the occurrence of cough. Duration of cough was also recorded. All concomitant drug intake record was also included.

All data were managed using SPSS for Windows version 20.0 (IBM Corp., Armonk, NY). Mean was calculated for continuous data and frequencies were calculated for categorical data. Chi-square was applied for comparison of categorical variables. P-value ≤0.05 was taken as significant.

## Results

A total of 123 participants taking ACEi for at least one month were included in this study. Their mean age was 58 ±13 years and there were more men than women (60% vs. 40%). Their demographics and clinical characters are shown in Table 1.

**Table 1:** Demographic and clinical characteristics of study participants ACEi - angiotensin-converting enzyme inhibitors; ARBs - angiotensin receptor blockers; BMI - body mass index; H2RA, H2 - receptor antagonists; OHA - oral hypoglycemic agents; PPIs - proton pump inhibitors; SD - standard deviation

Baseline characteristics	Frequency (%)
Age, years	
Mean ± SD	58 ±13
< 45	49 (39.8%)
≥ 45	74 (60.2%)
Gender	
Male	83 (67.5%)
Female	40 (32.5%)
BMI, kg/m^2^	
Mean ± SD	30.8 ± 5.8
< 30	51 (41.5%)
≥ 30	72 (58.5%)
Smokers	80 (65.0%)
ACEi therapy	
Enalapril	58 (47.2%)
Captopril	28 (22.7%)
Lisinopril	23 (18.7%)
Ramipril	14 (11.4%)
Duration of ACEi use in years	
Mean ± SD	2.5 ± 1.8
< 2	81 (65.8%)
≥ 2	42 (34.1%)
Concomitant drug usage	
None	25 (20.3%)
PPIs / H2RA	98 (79.7%)
OHA	68 (55.3%)
Nitrates	34 (27.6%)
Statins	47 (38.2%)
ARBs	27 (21.6%)
Diuretics	75 (60.9%)
Cardioselective B-blocker agents	71 (57.7%)
Calcium channel blockers	38 (30.8%)
Platelet aggregation inhibitors	69 (56.1%)
Co-morbidity status	
Cardiovascular diseases	105 (85.4%)
Diabetes mellitus type II	79 (64.2%)
Musculoskeletal disease / arthritis	44 (35.7%)
Stroke	38 (30.8%)
Neurological disease	27 (21.9%)
Genitourinary / nephrological disease	11 (8.9%)

As seen in Table 1, enalapril was the most frequently prescribed ACEi in this study followed by captopril, lisinopril, and ramipril was the least common. The demographic characteristics and ACEi-related characteristics were then classified according to the type of ACEi as shown in Table 2. Neither age nor gender of the participants were related to the type of ACEi causing cough. Patient’s BMI was statistically related (p=0.002). Smoking status was associated with the type of ACEi causing cough (p=0.008). Duration of ACEi usage and duration of cough was not related. Time of the day the pill was taken was also not statistically related to the type of ACEi causing cough. Time from the start of ACEi to the occurrence of cough was related to the type of ACEi prescribed (p=0.04). All parameters are shown in Table 2.

**Table 2:** Relationship of demographic and clinical characteristics of the participants with the type of ACEi causing cough ACEi - angiotensin-converting enzyme inhibitors; ARBs - angiotensin receptor blockers; HTN - hypertensive; BMI - body mass index

Characteristics	Enalapril (n=58; 47.2%)	Captopril (n=28; 22.7%)	Lisinopril (n=23; 18.7%)	Ramipril (n=14; 11.4%)	p-value
Age, years					
< 45	27 (46.6%)	10 (35.7%)	7 (30.4%)	5 (35.7%)	0.52
≥ 45	31 (53.4%)	18 (64.3%)	16 (69.5%)	9 (64.3%)	
Gender					
Male	41 (70.7%)	18 (64.3%)	15 (65.2%)	9 (64.3%)	0.91
Female	17 (29.3%)	10 (35.7%)	8 (34.7%)	5 (35.7%)	
BMI, kg/m^2^					
< 30	14 (24.1%)	17 (60.7%)	13 (56.5%)	7 (50%)	0.002
≥ 30	44 (75.8%)	11 (39.3%)	10 (43.5%)	7 (50%)	
Smoking status					
Smokers	34 (58.6%)	23 (82.1%)	18 (78.3%)	5 (35.7%)	0.008
Duration of ACEi use, years					
< 2	36 (62.1%)	20 (71.4%)	15 (65.2%)	10 (71.4%)	0.81
≥ 2	22 (37.9%)	8 (28.5%)	8 (34.7%)	4 (28.5%)	
Duration of cough, months					
< 2	35 (60.3%)	15 (53.5%)	18 (78.3%)	8 (57.1%)	0.30
≥ 2	23 (39.6%)	13 (46.4%)	5 (21.7%)	6 (42.9%)	
Time from start of ACEi to occurrence of cough, months					
< 1	13 (22.4%)	7 (25%)	10 (43.5%)	8 (57.1%)	0.04
1-3	20 (34.5%)	10 (35.7%)	9 (39.1%)	5 (35.7%)	
> 3	25 (43.1%)	11 (39.3%)	4 (17.4%)	1 (7.1%)	
Time of the day ACEi is taken					
Morning only	21 (36.2%)	12 (42.8%)	8 (34.7%)	5 (35.7%)	0.97
Bedtime only	20 (34.4%)	10 (35.7%)	7 (30.4%)	5 (35.7%)	
Morning and bedtime	17 (29.3%)	6 (21.4%)	8 (34.7%)	4 (28.5%)	
Concomitant Anti-HTN therapy					
None	18 (31.0%)	13 (46.4%)	10 (43.5%)	5 (35.7%)	Not applicable
ARBs	11 (18.9%)	-	2 (8.7%)	-	
Diuretics	9 (15.5%)	5 (17.8%)	5 (21.7%)	4 (28.5%)	
B-blockers	10 (17.2%)	6 (21.4%)	2 (8.7%)	2 (14.3%)	
Calcium channel blockers	10 (17.2%)	4 (14.3%)	4 (17.4%)	3 (21.4%)	

**Figure 1:**
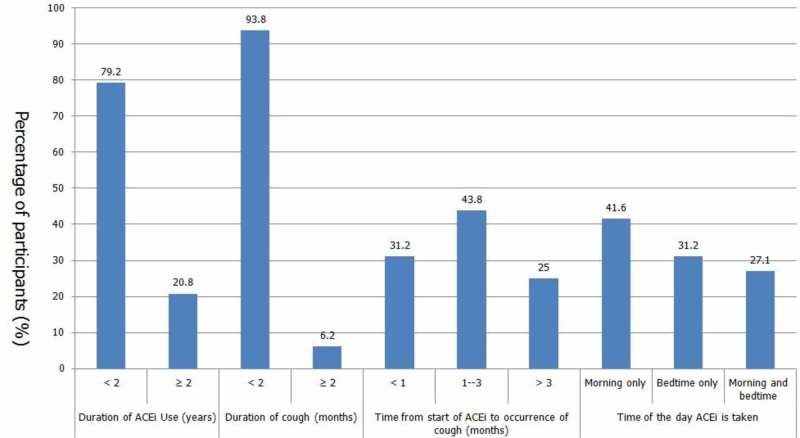
ACEi related characteristics of patients using ACEi as their only medication ACEi - angiotensin-converting enzyme inhibitors

There were 48 (39%) participants who were taking ACEi only and no other concomitant drug. Their ACEi related characteristics are shown in Figure 1.

When the patients were asked if their cough is significant enough to stop taking their ACEi, 33 (26.8%) responded that they plan to request their physician to prescribe them an alternate medication.

## Discussion

In this study, it was deduced that among the ACEi users presenting with cough, enalapril was the most commonly prescribed ACEi. Higher BMI, smoking, and longer time from the start of ACEi to the occurrence of cough are related to ACEi-induced cough.

When Haldberg et al. studied 121 patients of ACEi-induced cough, they found that enalapril was the culprit in 88% cases. As compared to our results, they did not find a statistical relationship of ACEi-induced cough with longer time to onset of cough and smoking [5]. In an analysis of three large trials on perindopril-induced cough, the rate of discontinuation was 4%. Significant determinants of cough included female gender, old age (>65 years), and simultaneous use of lipid-lowering agents [8]. In a large cohort, there were two and a half times higher risk of ACEi-induced cough in patients with a history of smoking and in East Asians patients. A twelve times higher risk in patients who have experienced cough with any other ACEi. ACEi discontinuation rate due to cough was 19% [11]. In this study, 27% of participants planned to request discontinuation of therapy to their physician. In a Singaporean report, the rate of therapy discontinuation due to ACEi-induced cough was 30% [13]. In another cohort of 1,700 patients taking ACEi, 12% developed a cough. The determinants of cough in this cohort were older age, female gender, non-African American ethnicity, ACEi naivety, and previous history of cough due to any other ACEi [12].

There has only been one report of ACEi-induced cough from Pakistan. They reported an overall 7% incidence of cough with ACEi use which was highest with lisinopril (9%). They did not report any data on enalapril which was the most common triggering agent in our report. The incidence of ramipril-related cough was 6.8% in their report [10]. In our analysis, 11% of ACEi-induced cough was due to ramipril. In a report from Poland, the incidence of ramipril-related cough was 7%. The factors determining the risk of cough in the Polish report were female gender, smoking, and respiratory illnesses including asthma, chronic obstructive pulmonary disease, and tuberculosis [14]. In a prospective study from Bahrain, 37% developed a perindopril-induced cough after the mean duration of onset of 12.7 days. After stopping or changing the drug, the mean for cough disappearance was 13.3 days. The ACEi-induced cough was related to female gender and not related to age or BMI [15]. In our study, BMI was a statistically significant determinant.

To the best of our knowledge, the characteristics of ACEi-induced cough have been studied for the first time in this study in Pakistani population. It was an observational study and controlled trials can be better utilized for more generalized results. The concept of pharmacovigilance (PV) is pretty much non-existent in the current pharmaceutical industry of Pakistan [16]. In order to comprehend the impact of ACEi-induced cough and other adverse drug reactions on the population, designing and implementing PV programs has become a grave necessity. Reports from country-wide PV records will give a more wholesome perspective of the situation.

## Conclusions

Despite their common utilization in hypertension and heart failure, adverse events associated with ACE inhibitors remain a major challenge for the physicians. The ACEi-induced cough may be severe enough to push the patients towards the discontinuation of therapy in some cases. Enalapril was most commonly associated with cases of ACEi-induced dry cough. Patient factors determining higher risk included high BMI, smoking, and longer time to onset of cough from the start of ACEi usage.

## References

[1] Rasmussen ER, Pottegård A, Bygum A, von Buchwald C, Homøe P, Hallas J (2019). Angiotensin II receptor blockers are safe in patients with prior angioedema related to angiotensin-converting enzyme inhibitors - a nationwide registry-based cohort study. J Intern Med.

[2] Rasmussen ER, von Buchwald C, Wadelius M (2017). Assessment of 105 patients with angiotensin-converting enzyme -inhibitor-induced angioedema. Int J Otolaryngol.

[3] McDowell SE, Coleman JJ, Ferner RE (2006). Systematic review and meta-analysis of ethnic differences in risks of adverse reactions to drugs used in cardiovascular medicine. BMJ.

[4] Caldeira D, David C, Sampaio C (2012). Tolerability of angiotensin - receptor blockers in patients with intolerance to angiotensin-converting enzyme inhibitors. Am J Cardiovasc Drugs.

[5] Hallberg P, Nagy J, Karawajczyk M (2017). Comparison of clinical factors between patients with angiotensin-converting enzyme inhibitor-induced angioedema and cough. Ann Pharmacother.

[6] Hallberg P, Persson M, Axelsson T (2017). Genetic variants associated with angiotensin-converting enzyme inhibitor-induced cough: a genome-wide association study in a Swedish population. Pharmacogenomics.

[7] Bangalore S, Kumar S, Messerli FH (2010). Angiotensin-converting enzyme inhibitor-associated cough: deceptive information from the Physicians' Desk Reference. Am J Med.

[8] Brugts JJ, Arima H, Remme W (2014). The incidence and clinical predictors of ACE-inhibitor induced dry cough by perindopril in 27,492 patients with vascular disease. Int J Cardiol.

[9] Sadanaga T, Yoshimura M, Sakamoto T, Sumida H, Ogawa H (2009). Enalapril-induced cough is associated with non-severe heart failure. Int J Cardiol.

[10] Nazir A, Sheikh FM, Aslam S, Javaid U (2016). Ace inhibitors; comparison of ace inhibitors (perindopril, ramipril & lisinopril) induced cough. Professional Med J.

[11] Morimoto T, Gandhi TK, Fiskio JM (2004). An evaluation of risk factors for adverse drug events associated with angiotensin‐converting enzyme inhibitors. J Eval Clin Pract.

[12] Morimoto T, Gandhi TK, Fiskio JM (2004). Development and validation of a clinical prediction rule for angiotensin-converting enzyme inhibitor-induced cough. J Gen Intern Med.

[13] Ng LP, Goh PS (2014). Incidence of discontinuation of angiotensin-converting enzyme inhibitors due to cough, in a primary healthcare centre in Singapore. Singapore Med J.

[14] Wyskida K, Jura-Szołtys E, Smertka M, Owczarek A, Chudek J (2012). Factors that favor the occurrence of cough in patients treated with ramipril-A pharmacoepidemiological study. Med Sci Monit.

[15] Nasser J, Aradi KA, Ebrahim KS, Omran A (2018). High incidence of cough among users of angiotensin-converting enzyme inhibitors. Bahrain Med Bull.

[16] Mahmood KT, Amin F, Tahir M, Haq IU (2011). Pharmacovigilance-a need for best patient care in Pakistan. A review. J Pharm Sci Res.

